# Investigating the Role of sHLA-G in Immunopathogenesis and Predicting Gastroesophageal Reflux Disease

**DOI:** 10.34172/mejdd.2025.415

**Published:** 2025-04-30

**Authors:** Wafaa Hazim Salih, Huda Saleem Hantoosh Hameed Al-khalidy, Batool Mutar Mahdi, Laith Hikmet Muhsun

**Affiliations:** ^1^Department of Microbiology, Al-Kindy College of Medicine, University of Baghdad, Baghdad, Iraq; ^2^Department of Biochemistry, Al-Kindy College of Medicine, University of Baghdad, Baghdad, Iraq; ^3^HLA Research Unit, Al-Kindy College of Medicine, University of Baghdad, Baghdad, Iraq; ^4^Al-kindy Teaching Hospital, Baghdad, Iraq

**Keywords:** GERD, sHLA-G, Esophagus

## Abstract

**Background:**

Gastroesophageal reflux disease (GERD) is a chronic gastrointestinal disease in which the patients may be asymptomatic or complain of heartburn and regurgitation or pulmonary symptoms. Aim of the study: Examine the serum level of soluble HLA-G (sHLA-G) in patients with GERD, which can be used as a biomarker for early detection of GERD.

**Methods:**

The study was a case-control prospective study that enrolled 40 patients who consulted the Gastroenterology Unit-Al-Kindy Teaching Hospital and were diagnosed with GERD by their physician. They were compared with 40 control healthy individuals from January 2023 to May 2024. Serum samples were used for quantitative assessment of sHLA-G using a sandwich enzyme-linked immunosorbent assay.

**Results:**

A higher proportion of females were observed in the GERD group compared with the control group (72.5% vs. 42.6%). This difference was statistically significant (*P*=0.006). A statistically non-significant difference was observed in sHLA-G levels between patients with GERD and healthy controls (*P*=0.158). The median sHLA-G level was non-significantly higher in patients with GERD (0.529 ng/mL) compared with the controls (0.523 ng/mL).

**Conclusion:**

The present study provides early evidence for an association between elevated sHLA-G levels and GERD with limited diagnostic value. It revealed a significant negative correlation between sHLA-G levels and sex. Further studies are needed to elucidate the mechanisms underlying this relationship and explore the potential of sHLA-G as a diagnostic or therapeutic target for GERD.

## Introduction

 Gastroesophageal reflux disease (GERD) is a condition characterized by abnormal gastric content reflux or regurgitation into the esophagus at least once a week, leading to heartburn and epigastric pain.^1^ It is a common disease worldwide, with increasing frequency rates of GERD in developed and developing countries.^2^ Meanwhile, risk factors like genetics, age, sex, obesity, and lifestyle changes have led to an increasing frequency of symptomatic GERD.^3^ Moreover, poor lifestyle habits and behaviors can worsen GERD symptoms and decrease treatment efficacy.^4^ The main clinical symptoms of GERD include heartburn, epigastric pain, dyspepsia, and extraesophageal symptoms like chronic cough, pharyngitis, and asthma.^5^ Reflux esophagitis may progress to Barrett’s esophagus and esophageal adenocarcinoma with high morbidity and mortality.^6^ So, early diagnosis is important to prevent these complications, which can be achieved via clinical symptoms, upper endoscopy that uses a tiny camera on the end of a flexible tube, and PH monitoring using a catheter or camera.^7^ Genetic factors may be used as biomarker sensors for early detection of GERD. Both genetic variations, like alleles of classical human leukocytes antigens (HLA) (HLA-DRB1 *15:01), and FOX1 gene that affects the growth of stomach smooth muscle may increase the chance of developing GERD.^8,9^ Other non-classical HLA, like soluble HLA-G (sHLA-G) can be a potential diagnostic biomarker for some diseases due to its suppressive immune function and over-expression of this soluble molecule in some diseases with the influence of cytokines production.^10^ In the present study, we sought to examine the serum level of sHLA-G in patients with GERD, which can be used as a biomarker for the early detection of GERD.

## Materials and Methods

 The design of the study was a case-control prospective enrolled 40 patients consulted Gastroenterology Unit, Al-Kindy Teaching Hospital, were diagnosed with GERD by their physician, and compared to the second 40 control healthy group from 15 January 2023 to 20 May 2024. The inclusion criteria were patients of different sexes who complained of mild esophageal symptoms like heartburn, regurgitation, dyspepsia, dysphagia, and epigastric pain. GERD diagnosis was confirmed via upper gastroesophageal endoscopy using gastroscope: GIFH260; Olympus, Tokyo, Japan, and Display screen; Olympus OEV-261H liquid crystal display monitor; Olympus, Tokyo, Japan. Only patients with grade B were selected and included in this study. The classification was done according to the Los Angeles classification of GERD, 2013, as follows: Grade A: One (or more) mucosal break no longer than 5 mm that does not extend between the tops of two mucosal folds; Grade B: One (or more) mucosal break more than 5 mm long that does not extend between the tops of two mucosal folds, Grade C: One (or more) mucosal break that is continuous between the tops of two or more mucosal folds but which involve less than 75% of the circumference, and Grade D: One (or more) mucosal break which involves at least 75% of the esophageal circumference.^11^

 The Exclusion criteria were patients with tumors of the esophagus, stomach, and intestine, esophageal varicose veins, *Helicobacter pylori *test positive, or currently using drugs like proton pump inhibitors (PPIs), antacids, glucocorticosteroids, non-steroid anti-inflammatory drugs, H2-histamine receptor blockers, calcium channel antagonist, and nitrates.

 Demographic data were obtained from both groups, including age, sex, weight, height, body mass index (BMI), smoking, and address. Other detailed patient histories included lifestyle and dietary habits, other members of the family having the same disease, and any genetic-related factors made during participant selection.

 Venous blood was aspirated, and serum was separated for assessment of soluble HLA-G (sHLA-G) using a sandwich enzyme-linked immunosorbent assay (ELISA) kit for the quantitative evaluation of total sHLA-G in serum of both groups according to the instructions of manufacturer kit (Cat. No YLA1602HU, Biont, Bioassay Technology Laboratory, China).

###  Statistical Analysis

 The findings were collected using Excel and analyzed using Medcalc and SPSS software version 25.0. Continuous variables were calculated and expressed as mean ± standard error of the mean (SEM), then analyzed using student’s *t* test to assess the level of significance. Other categorical variables were expressed as numbers and percentages and analyzed using another test, the χ^2^ test, with a 95% confidence interval (CI). Receiver operating characteristic (ROC) curve analysis was used to assess the area under the curve (AUC), 95% CI, cut-off value, sensitivity, specificity, positive predictive value, negative predictive value, and accuracy. Pearson and Spearman correlation analysis was applied to determine the correlation coefficient between different variables. Statistical significance was *P* ≤ 0.05.

## Results

**Figure 1:**
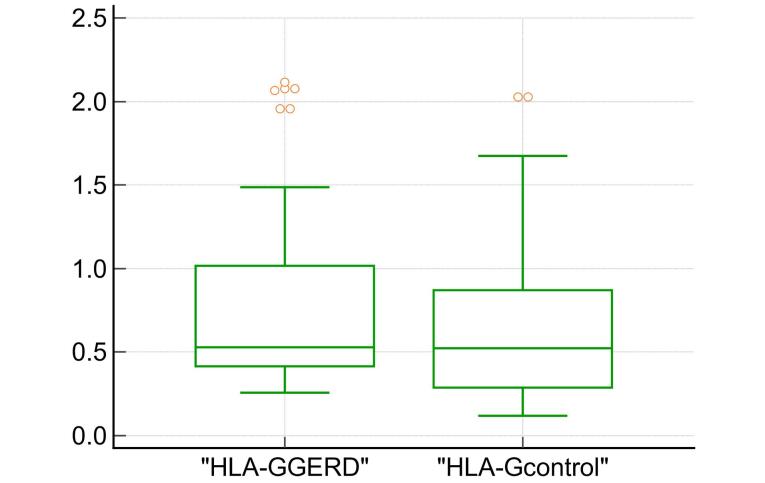
Median level of sHLA-G of patients with GERD and controls

 The demographic characteristics of the patients with GERD and controls are presented in Table 1. A higher proportion of females was observed in the GERD group compared with the control group (72.5% vs. 42.6%). This difference was statistically significant (*P* = 0.006). Additionally, patients with GERD were significantly older than healthy controls (median age 42 vs. 28). Regarding obesity and smoking as risk factors, patients with GERD had a significantly higher mean BMI compared with healthy controls (24.8 kg/m^2^ vs. 22.3 kg/m^2^), and a higher proportion of patients with GERD were smokers compared with the controls (62.5% vs. 35%). A statistically non-significant difference was observed in sHLA-G levels between patients with GERD and healthy controls (*P* = 0.158) (Figure 1). The median sHLA-G level was non-significantly higher in patients with GERD (0.529 ng/mL) compared with the controls (0.523 ng/mL).

**Table 1:** Demographic characteristics and sHLA-G levels in patients with GERD and controls

**Characteristics variables **	**Patients with GERD (n=40)**	**Healthy controls (n=40)**	**95% CI**	***P*** ** value**
Sex (Male), No. (%)	11 (27.50)	23 (57.40)	0.3151-0.5406	0.006*
Sex (Female), No. (%)	29 (72.50)	17 (42.60)	0.4594-0.6849	
Address -Baghdad, No. (%)	40 (100)	40 (100)	0.00-0.08	0.0
Age (years), Mean ± SEM	43.93 ± 2.87 (15-85)	34.45 ± 3.13 (11-76)	1.36-17.59	0.023**
Smoking positive	25 (62.5%)	14 (35%)		0.013*
BMI kg/m^2^, Mean ± SEM	24.799 ± 0.36 (17-31)	22.27 ± 0.671 (14-29)		0.001**
sHLA-G (Median)	0.529	0.523	-0.021 to 0.264	0.158***
Maximum	2.116	2.028		
Minimum	0.257	0.119		
sHLA-G - Q1	0.415	0.282		
sHLA-G - Q3	1.030	0.973		
sHLA-G - IQR	0.615	0.691		

*Significant (χ^2^ test); **Significant (student’s t-test). ***Non Significant (Mann-Whitney test)

**Figure 2:**
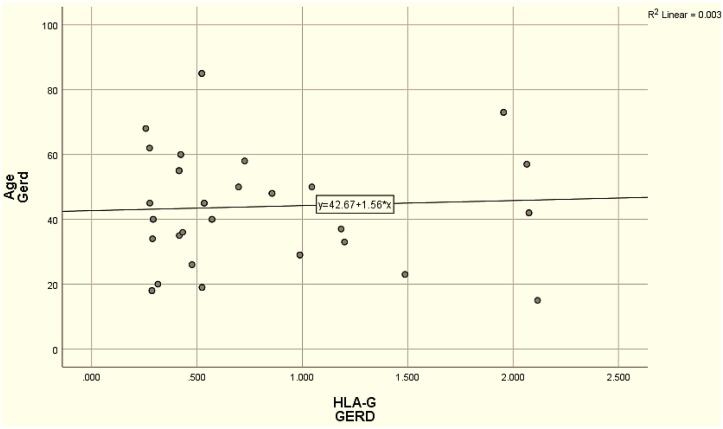
Pearson correlation analysis between sHLA-G and age in patients with GERD

**Figure 3:**
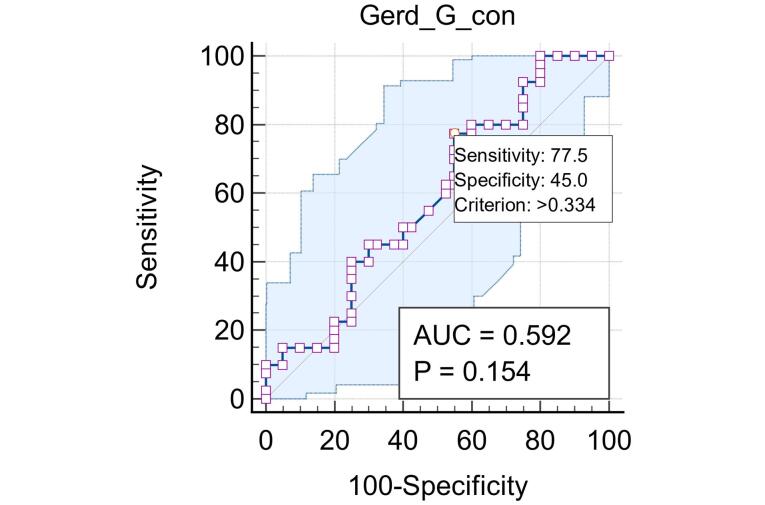
Receiver operating characteristic (ROC) curve plot of sHLA-G for predicting GERD (Area under curve = 0.592 (95%CI = 0.476 to 0.701) (*P* = 0.154), SEM = 0.064, Cut-off value = < 0.334, Sensitivity = 77.5%, Positivity = 45.0%, Positive predictive value = 58.5, Negative predictive value = 66.7, Accuracy = 0.225.

 Concerning the correlation analysis Between sHLA-G and other variables, Pearson and Spearman correlation analyses were conducted to assess the relationship between sHLA-G levels and demographic variables (age, BMI, sex, and smoking) in patients with GERD. A significant negative correlation was observed between sHLA-G levels and sex (r = -0.619, *P* < 0.01), while no significant correlations were observed between sHLA-G levels and age or BMI. A weak positive correlation was found between sHLA-G and smoking (r = 0.157), but it was not statistically significant (Table 2, Figure 2). The diagnostic performance of sHLA-G in predicting GERD was done via ROC curve analysis, and the area under the ROC curve (AUC) was calculated to evaluate the discriminatory ability of sHLA-G. AUC was 0.592 (95% CI: 0.476-0.701, *P* = 0.154), and the optimal cut-off value for sHLA-G was determined to be ≤ 0.334, sensitivity 77.5%, specificity 45.0%, positive predictive value 58.5%, negative predictive value 66.7%, and accuracy 22.5% (Figure 3).

**Table 2:** Pearson and Sperman’s correlation analysis between sHLA-G and other variables in patients with GERD

**Variables **	**sHLA-G patients (pg/mL) (N=40)**	**Age (y)**	**BMI (kg/m**^2^**)**	**Sex**	**Smoking**
sHLA-G patients (pg/mL) (N = 40)	1	0.054	-0.091	-0.619**	0.157

**Correlation is significant at the 0.01 level (2-tailed).

## Discussion

 This study hypothesizes that sHLA-G, due to its role in immune modulation, may influence inflammatory pathways or immune responses within the esophageal mucosa, potentially contributing to the pathogenesis of GERD or its complications. The primary motivation behind our study is to explore a potential yet under-researched aspect of GERD pathophysiology. While sHLA-G has been widely studied in the context of immune regulation, particularly in cancer and autoimmune diseases, its role in GERD has not been extensively explored. However, GERD is increasingly recognized as a condition with significant immune-mediated components involving inflammation and mucosal injury in the esophagus. sHLA-G is known to modulate immune responses by inhibiting the activation of T cells and promoting immune tolerance, which could theoretically play a role in regulating the inflammatory processes that contribute to GERD development and its progression. This study included patients with GERD (grade B) who did not take medications like PPIs and corticosteroids to minimize potential confounding effects that could skew the interpretation of sHLA-G levels. Both these medications have known immune-modulating properties. PPIs, for example, can alter gastric acid secretion and have been shown to influence immune responses, potentially affecting the levels of immune markers like sHLA-G. Similarly, glucocorticosteroids are potent immunosuppressive agents that can significantly alter immune system function, potentially reducing sHLA-G levels or influencing its expression. So, the findings of this study suggested that patients with GERD are more likely to be female, older, have higher BMIs, and smoke compared with healthy controls. Additionally, elevated sHLA-G levels might be associated with GERD. The potential explanation may be due to hormonal factors, and differences in lifestyle behaviors among sexes might contribute to the higher prevalence of GERD in females. Anatomical changes with advanced age (more than 50 years), like changes in the lower esophageal sphincter tone, alteration in esophageal motility, and delayed gastric emptying, can increase the risk of GERD. Other factors like over-weight, obesity, increased BMI, and smoking are established risk factors for GERD, and their higher prevalence in the GERD group aligns and is in agreement with previous studies that reported increased frequency of GERD with aging^12^ menopausal women,^13^ increased intra-abdominal pressure, obesity,^14^ and smoking.^15^ Regarded sHLA-G showed a non-significant mild elevation in sHLA-E levels in patients with GERD, which might reflect an altered immune response or be involved in the pathogenesis of the disease and the grade of the GERD (grade B), which may be the inflammation still in early stage and this need follow-up the patients to assess the level of sHLA-G in advanced grades (grades C and D). To our knowledge, no articles have reported the association between sHLA-G and GERD. Other studies reported the association between sHLA-G and malignancy and that it might be a potential biomarker for tumors like gastrointestinal, colorectal, lung, breast, and ovary.^16^ So, further research is needed to evaluate the clinical utility of sHLA-G in the diagnosis and management of GERD. Regarding correlation, there was a significant negative correlation between sHLA-G levels and sex, suggesting a potential association between sex and the expression of sHLA-G in patients with GERD, which may be due to hormonal factors or other sex-related differences might influence the expression of sHLA-G in patients with GERD. However, further studies are required to confirm the nature of this association. The lack of significant correlations between sHLA-G levels in patients with GERD and age or BMI indicates that these factors might not be major determinants or causes of sHLA-G expression in patients with GERD. Limited or no studies are available on the correlation between sHLA-G levels and demographic variables in patients with GERD. However, studies have explored the role of HLA-G in other diseases like gastric cancer, which illustrates the association between HLA-G expression and disease severity.^17^ Other studies indicated that sHLA-G can be a potential diagnostic biomarker for head and neck cancer due to its suppressive function of this molecule and over-expression in diseased patients with the influence of cytokines.^18^ Further studies are needed to compare these results with the current study and to elucidate the specific role of sHLA-G in GERD pathogenesis. The ROC curve analysis indicated that sHLA-G had limited and restricted discriminatory ability in predicting and diagnosing GERD, as evidenced by the AUC of 0.592 and the non-significant *P* value of 0.154. While the sensitivity and negative predictive value are moderately high, the specificity and positive predictive value are relatively low. This suggests that sHLA-G might not be a highly accurate or clinically useful biomarker for GERD diagnosis. To our knowledge, no studies dealt with the diagnostic performance of sHLA-G for GERD. However, studies have explored the role of HLA-G in other inflammatory diseases like Crohn’s disease.^19^ So, HLA-G could be useful in predicting the risk of complications such as Barrett’s esophagus and esophageal adenocarcinoma, used as a biomarker for identifying patients who may be at higher risk for these complications, which could aid in surveillance and early intervention strategies. Additionally, there is an association between HLA-G and cancer.^20^

 The limitation of the study was that it had a small sample size, which needs further research with larger sample sizes and follow-up patients to validate and confirm these findings and explore the clinical utility of sHLA-G in diagnosing and managing GERD.

## Conclusion

 The present study provides early evidence for an association between elevated sHLA-G levels and GERD with limited diagnostic value and revealed a significant negative correlation between sHLA-G levels and sex. Further studies are needed to elucidate the specific mechanisms underlying this relationship and to explore the potential of sHLA-G as a diagnostic or therapeutic target for GERD.
